# Effect of Enhanced External Counterpulsation (EECP) on Exercise Time Duration and Functional Capacity in Patients with Refractory Angina Pectoris

**Published:** 2014-01-12

**Authors:** Ali Bozorgi, Entezar Mehrabi Nasab, Akram Sardari, Mostafa Nejatian, Shaghayegh Nasirpour, Sakineh Sadeghi

**Affiliations:** *Tehran Heart Center, Tehran University of Medical Sciences, Tehran, Iran.*

**Keywords:** *Angina pectoris* • *Counterpulsation* • *Echocardiography* • *Exercise test*

## Abstract

***Background:*** Enhanced external counterpulsation (EECP) is a noninvasive technique used for patients with refractory angina pectoris. There are controversial data on the effectiveness of EECP in improving patients with refractory stable angina. The aim of the present study was to evaluate the effectiveness and safety of EECP for the treatment of patients with refractory angina pectoris.

***Methods:*** Twenty consecutive patients with refractory angina pectoris were treated with EECP, and their symptoms, echocardiographic measures, treadmill exercise test parameters, and Canadian Cardiovascular Society Class were evaluated before and immediately after EECP. The patients were followed up for 6months post treatment.

***Results:*** There were significant differences regarding total exercise time before and after treatment (p value < 0.001). The patients showed a significant reduction in angina classes III and IV immediately after EECP (p value < 0.001); for most of the patients, these beneficial effects were sustained for 6 months (p value = 0.010). There was no significant improvement in the echocardiographic parameters.

***Conclusion: ***EECP decreased symptoms and increased total exercise time in our study population. These beneficial effects were sustained for 6 months.

## Introduction

Refractory angina pectoris is a clinical diagnosis characterized by chronic angina due to coronary artery disease in patients refractory to the conventional forms of treatment.^1^ The treatment of coronary artery disease consists of pharmacological intervention and invasive procedures such as percutaneous coronary intervention (PCI) and coronary artery bypass grafting (CABG). In spite of these generally successful means of treatment, the number of patients with severe symptomatic ischemic chest pain has increased.^2^

It has been reported that up to 15% of patients with angina pectoris meet the criteria for refractory angina (Mannheimer C. Therapeutic challenges of refractory angina pectoris. In XXth congress of the European society of Cardiology, Vienna, Austria 1998.). Enhanced external counterpulsation (EECP) is a noninvasive counterpulsation technique employed for the treatment of patients with chronic stable angina pectoris.^3^ The basic principle of EECP is the augmentation of the diastolic arterial pressure along with increasing the venous return. The hemodynamic effects of EECP are similar to those of intra-aortic balloon pumping, but the treatment yields more long-lasting effects.^4^ The mechanism underlying the effects of EECP is under investigation; however, several theories have been postulated.^3^ The hemodynamic effects of EECP enhance cardiac output, stroke volume, and retrograde aortic diastolic flow. Moreover, EECP produces hemodynamic changes that reduce myocardial oxygen demand in addition to potential for increased transmyocardial pressure to open collaterals. With exposure to the augmented blood flow and endothelial shear stress, there is elaboration of nitric oxide, prostacyclin, and vascular endothelial growth factor from the arterial bed, which improves endothelial function and vascular remodeling.^5^ EECP can improve angina, quality of life, exercise tolerance, time to ST-segment depression at rest and during stress, and Dobutamine stress-induced regional wall motion abnormality in patients with refractory angina.^6^ The beneficial effects of EECP can be achieved immediately after a course of treatment with long-term sustained improvement in angina control and quality of life.^7^

The aim of the present study was to evaluate the effect of EECP on patients with refractory angina pectoris. The study was designed to examine the immediate and 6-month follow-up effects on patients with severe refractory angina in whom multiple efforts such as CABG and PCI had already been made and where further medical and surgical interventions were exhausted.

## Methods

The EECP device consists of three paired pneumatic cuffs applied to the lower extremities. Patients are typically treated for a one-hour daily program for a total of 35 sessions over 7 weeks. The three sets of pneumatic cuffs are wrapped around the patient’s calves, lower thighs, and upper thighs and are inflated sequentially at the onset of diastole, and 300 mm Hg of external pressure is applied during diastole. At the onset of systole, the external pressure in the cuffs is released, producing a decrease in systolic pressure. A computer-controlled pneumatic system with a display console is employed to inflate and deflate the series of compressive cuffs, and inflation and deflation are triggered by events in the cardiac cycle through microprocessor-interpreted electrocardiograph signals. The compression is triggered by the electrocardiographic R-wave, with the delay being adjusted until the induced retrograde pulse wave enhances the cardiac output as reflected by an optimally augmented blood pressure wave during cardiac diastole. A finger plethysmogram is utilized throughout the treatment to monitor the diastolic and systolic pressure wave form. By setting the inflation and deflation timing, an optimal hemodynamic effect is obtained with the plethysmogram.

The study population was comprised of 20 patients. The patients were diagnosed with refractory stable angina pectoris despite optimized medical (pharmacological) and invasive therapies. Medical therapies included the maximally tolerated use of anti-angina medication.

The patients’ data, which had been recorded prior to treatment, consisted of age, gender, body weight, past history of diabetes mellitus, hypertension, dyslipidemia, cigarette smoking, and previous angiographic and surgical information, if available.

Three patients had a history of hypertension, 6 patients had hyperlipidemia, 5 patients had diabetes mellitus, 16 patients had significant coronary artery disease, 4 patients were active smokers, one patient had peripheral vascular disease, and finally renal function was normal in all the patients.

These patients were referred for EECP due to refractory stable angina pectoris despite pharmacological therapy and were considered to be unsuitable for or unwilling to consider revascularization by conventional percutaneous or surgical treatments. All the patients had documented coronary artery disease (coronary angiography or history of myocardial infarction).

All the patients were assessed by a cardiologist prior to EECP. Relevant background medical history was recorded. Echocardiography was performed to assess left ventricular (LV) function and exclude significant aortic regurgitation. An abdominal ultrasound was done to exclude clinically significant abdominal aortic aneurysm, and Doppler sonography of the lower limbs was performed to exclude deep vein thrombosis. The Canadian Cardiovascular Society (CCS) angina grading, angina frequency, and use of short-acting glyceryl trinitrate (GTN) in the weeks prior to EECP were recorded. All major events, including death, myocardial infarction, unstable angina, PCI, CABG, decompensated heart failure, new dysrhythmias, and repeat EECP and hospitalization due to a cardiac cause during the treatment period and follow-up were also recorded.

EECP per se is a noninvasive and safe therapy with minimal complication if the patient is selected properly. One of our patients had non-advanced peripheral arterial disease. Accordingly, there was no complication related to the therapy itself in our patients.

Echocardiography was performed before and after EECP. The parameters which were recorded included LV end-diastolic and end-systolic diameters (LVEDD and LVESD), LV ejection fraction (LVEF), regurgitation of the mitral and other valves, right ventricular (RV) size and function, and LV diastolic function. 

Before and after EECP, the symptom-limited Modified Bruce exercise treadmill test was performed. Original copies of all exercise tests and investigator data sheets were examined by a single physician, who was blinded to the data. ST-segment depression was made at a point 60 milliseconds after the J junction. A positive test result was defined as ≥1mm of additional horizontal or down-sloping STD. Heart rate recovery was calculated as the difference in the heart rate at peak exercise and at one minute and 2 minutes of recovery.

The descriptive data are presented as mean with standard deviation (SD) or median with interquartile range (IQR). The Wilcoxon or paired t-test was used to compare improvement in the echocardiographic parameters [LVEF, LVEDD, LVESD, right ventricular diameter (RVD), and left atrial size], exercise test parameters, metabolic equivalent (METs), total exercise time, heart rate recovery, maximum heart rate, maximum blood pressure, and the CCS functional class before and after EECP.

## Results

The study population consisted of 20 patients, whose baseline characteristics are depicted in Table 1. All the patients had advanced coronary artery disease, and most of them had prior myocardial infarction and coronary revascularization with percutaneous coronary intervention (PCI) and/or CABG and were, therefore, not good candidates for further conventional revascularization.

At study entry, echocardiography and exercise test parameters, the CCS function class, and quality of life were evaluated.

Changes before and after EECP were not statistically significant in terms of LVEF, LVEDD, and LVESD before and after EECP (Table 2). However, there were statistically significant changes between the exercise test duration (p value < 0.001), METs (p value = 0.001), and EF (p value = 0.012) before and after EECP (Table 2). Nevertheless, an increase of 2.7% for EF is not clinically important.

The median of heart rate recovery one minute after exercise increased from 7 beats/min (range = 1.2 to 15.2) to 9 beats/min (range = 1 to 20; p value = 0.274). This median value at 2 minutes after exercise increased from 22 beats/min (range = 9 to 31.7) to 26 beats/min (range =10 to 32.5; p value = 0.137).

The changes were not statistically significant with respect to systolic and diastolic blood pressure before and after EECP (Table 2).

**Figure 1:**
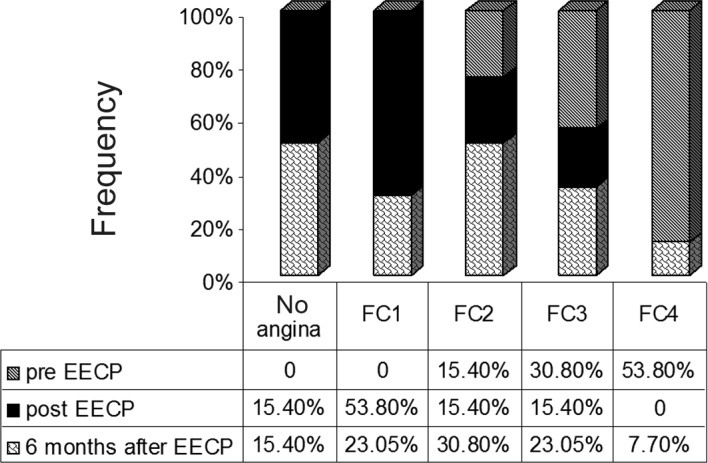
Comparison of angina Canadian Cardiovascular Society class pre enhanced external counterpulsation and post enhanced external counterpulsation and follow-up after 6 months

FC, Functional capacity; EECP, Enhanced external counterpulsation

The patients demonstrated a significant reduction in angina classes III and IV after EECP (p value < 0.001) and after 6-month follow-up (p value = 0.010) (Figure 1).

There was a decrease in the use of nitroglycerine, and it was sustained at 6-month follow-up.

**Table 1:** Patients’ demographics and baseline characteristics (n = 20)

Age (y)	61.50±10.13
Male sex	19
Hypertension	3
Hyperlipidemia	6
Diabetes mellitus	5
Smoking	4
Congestive heart failure	8
Prior PCI	3
Prior CABG	13
Peripheral vascular disease	1
Positive family history	4

PCI, Percutaneous coronary intervention; CABG, Coronary artery bypass grafting

**Table 2:** Comparison of variables before and after EECP

	Before EECP	After EECP	P value
Systolic blood pressure (mmHg)	119.40±12.59	116.65±12.18	0.348
			
Diastolic blood pressure (mmHg)	71.90±8.03	69.25±9.71	0.281
			
Heart rate (/min)	72.00±14.55	68.40±10.00	0.063
			
Weight (kg)	74.90±13.16	74.20±13.04	0.038
			
Echocardiography			
LVEF (%)	35.75±12.16	38.50±12.68	0.012
LVESD (mm)	39.70±8.98	40.05±7.38	0.796
LVEDD (mm)	55.30±7.26	54.15±6.93	0.282
			
Exercise test			
METs (mlo^2^/kg/min)	4.60 (3.40-6.90)	6.90 (4.60-9.20)	0.001
Total exercise time (min)	8.60 (5.60-10)	9.90 (8.10-12.70)	< 0.001
Recovery-1 (beat/min)	7 (1.20-15.20)	9 (1-20)	0.274
Recovery-2 (beat/min)	22 (9-31.70)	26 (10-32.50)	0.137
Max. heart rate (/min)	68.10±11.02	74.30±10.12	0.063
Max. systolic blood pressure (mmHg)	131.75±17.36	136.25±16.45	0.348
Max. diastolic blood pressure (mmHg)	79.50±2.61	79.25± 2.93	0.281

EECP, Enhanced external counterpulsation; LVEF, Left ventricular ejection fraction; LVESD, Left ventricular end-systolic diameter; LVEDD, Left ventricular end-diastolic diameter; MET, Metabolic equivalent; Max, Maximum

* Data are presented as mean±SD or median (interquartile range)

## Discussion

The aim of the present study was to evaluate the effectiveness and safety of EECP for the treatment of patients with refractory angina pectoris in our center. In this study, we confirmed that EECP significantly improved exercise tolerance, in concordance with the findings in MUST-EECP,^8^ and reduced angina. Our patients, who were predominantly in the CCS functional classes II, III, and IV before EECP, showed a sustained significant decrease in their weekly angina episodes and a dramatic reduction in the angina functional class immediately post treatment. In addition, nitroglycerine use remained significantly decreased compared with baseline, which chimes in with the results of some other studies.^7, 9-13^

In our study, a reduction in the functional class was seen in the patients with functional classes II, III, and IV, whereas in other studies, the beneficial effect was limited to patients with functional classes III and IV.^14^ Also in the present study, 33% of the patients who experienced a beneficial effect of EECP improved by three functional classes and 42% of the patients improved by two functional classes. The improvement by two functional classes tended to progress over a period of 6 months and was more prominent in the patients with functional class IV prior to the EECP treatment. 

Regarding treadmill exercise test parameters, the most interesting result was a significant increase in the total exercise time and METs. There was a trend toward improvement in heart rate recovery (in the first and second minutes after exercise). We believe that with an increase in the sample, the differences in this parameter before and after EECP would constitute statistical significance. 

The mechanisms whereby these hemodynamic effects alleviate refractory angina pectoris are still poorly understood. It is, however, believed that these effects are similar to those in intra-aortic balloon pumping.^4^

Immediate reduction in angina and increase in exercise tolerance can occur without any improvement in myocardial perfusion, and the early benefits of EECP may be attributed to peripheral effects similar to those of exercise training.^15^ The proposed mechanisms of sustained long-term benefits are multi-factorial and include improvement in the endothelial function, collateral recruitment and angiogenesis, exercise training effect, and neurohormonal modulation.^16-19^

Our study shows that most patients with angina refractory to conventional therapy report symptomatic relief and increase in their exercise capacity both in the short and long term, which will lead to a reduction in the need for pharmacological therapy. The International EECP Patient Registry (IEPR)^12^ reported sustained improvement in angina and quality of life in the majority of patients over a 2-year period. Furthermore, an observational study has reported that the benefits may be sustained for 5 years.^10^

In our study, the most beneficial effect of EECP was observed immediately after the procedure, and the effect was diminished in the months following the procedure. 

## Conclusion

Chiming in with other similar studies, there was a significant improvement in functional capacity, total exercise time, and METs after 35 sessions of EECP. Also, the improvement in functional capacity still persisted at 6 months after EECP. There was a statistically significant improvement in EF in the patients after EECP, but it was not clinically significant.

## Notes

***This paper should be cited as:*** Bozorgi A, Mehrabi Nasab E, Sardari A, Nejatian M, Nasirpour S, Sadeghi S. Effect of Enhanced External Counterpulsation (EECP) on Exercise Time Duration and Functional Capacity in Patients with Refractory Angina Pectoris. J Teh Univ Heart Ctr 2014;9(1): 33-37
